# The effect of simulating body fluid on the structural properties of hydroxyapatite synthesized in the presence of citric acid

**DOI:** 10.1007/s40204-016-0055-5

**Published:** 2016-10-05

**Authors:** Omer Kaygili, Serhat Keser, Mustafa Kom, Niyazi Bulut, Sergey V. Dorozhkin

**Affiliations:** 1 Department of Physics, Faculty of Science, Firat University, 23119 Elazig, Turkey; 2 Department of Chemistry, Faculty of Science, Firat University, 23119 Elazig, Turkey; 3 Department of Surgery, Faculty of Veterinary Medicine, Firat University, 23119 Elazig, Turkey; 4 Kudrinskaja sq. 1-155, Moscow, Russia 123242

**Keywords:** Hydroxyapatite, Citric acid, Bioceramics, Simulated body fluid

## Abstract

In present work, the effect of citric acid (CA) addition in different amounts (0, 1, 5 and 10 ml) on the structure of hydroxyapatite (HAp) was investigated using X-ray diffraction (XRD), Fourier transform infrared (FTIR) spectroscopy, scanning electron microscopy (SEM) and energy dispersive X-ray (EDX) spectroscopy techniques. The crystallite dimensions, lattice parameters, unit cell volume, crystallinity percentage and Ca/P molar ratio were found to be affected by the CA content. To investigate the influence of CA on the bioactive properties of the HAp samples and to determine the optimum amount of CA, in vitro soaking tests in simulated body fluid (SBF) were performed. Although the samples’ morphology was found to be affected by neither the amount of CA nor the soaking time in SBF, the soaking results revealed that the maximum changes in the Ca/P ratio were found for the HAp samples prepared in the presence of the highest amounts of CA, which pointed out to the highest bioactivity of these samples.

## Introduction

Due to its chemical and morphological similarity to the inorganic part of human hard tissues, non-toxicity and very high biocompatibility, hydroxyapatite (HAp, Ca_10_(PO_4_)_6_(OH)_2_) appears to be a very important and useful biomaterial suitable for biomedical applications (Suchanek and Yoshimura 1998; Sopyan et al. 2007; Dorozhkin 2012, 2016; Supova 2015; Kaygili et al. 2014, 2015). Therefore, many studies have been published on HAp preparation and investigation of its major properties. Namely, synthetic HAp can be successfully prepared using various experimental techniques, such as sol–gel (Sanosh et al. 2009), spray pyrolysis (Cho et al. 2016), solution combustion (Pratihar et al. 2006), hydrothermal (Manafi and Rahimipour 2011), microwave (Ruban Kumar et al. 2010), precipitation (Mobasherpour et al. 2007) and extraction from natural resources (Akram et al. 2014). Among them, sol–gel is an inexpensive method for preparing of the nano-sized HAp, having high crystallinity and purity, at low temperatures (Kalita et al. 2007; Kaygili and Tatar 2012). However, the chemically pure HAp appears to be a rather inert compound, which after implantation remains unchanged for many years; therefore, ways are sought to improve its bioactivity. Preparation of HAp in the presence of various additives appears to be the common way to solve this problem (Dorozhkin 2012, 2016; Supova 2015; Kaygili et al. 2014, 2015).

Citric acid (CA, C_6_H_8_O_7_) is an important substance used mainly in the pharmaceutical and food industries, as well as in detergents and cleaning products, cosmetics and toiletries (Soccol et al. 2006). In addition, CA is believed to affect the formation of human bones by adsorbing in both the reacting and the producing phases of calcium ions; therefore, there is a large interest in the synthesis of calcium phosphates in the presence of CA (Brecevic and Füredi-Milhofer 1979; Rhee and Tanaka 1999; Misra 1996; Weng et al. 2002; de Leeuw and Rabone 2007; Chang et al. 2011; Sun et al. 2014; Skwarek et al. 2014; Iafisco et al. 2015; Kaygili et al. 2015). Some of these investigations are mentioned in the chronological order as follows. Misra (1996) published a report related to the interaction of CA with synthetic HAp and investigated the surface exchange of ions and precipitation of calcium citrate on HAp. Weng et al. (2002) investigated the effect of CA addition on the gelation mechanism in HAp synthesized via the sol–gel route. Leeuw and Rabone investigated the effects of the adsorption of CA to two specific surfaces of HAp using molecular dynamic simulations. CA was found to attach to all surfaces of HAp crystals and inhibited their growth but in various extents (de Leeuw and Rabone 2007). The influence of CA on the formation, purity and particle size distribution of HAp was studied by Chang et al. The authors discovered that the content of CaO as an unavoidable major impurity was reduced due to the addition of CA (Chang et al. 2011). Sun et al. (2014) showed that the CA-based polymer/hydroxyapatite composite scaffolds improved the repair of rat calvarial defects. Skwarek et al. (2014) published a study related to the adsorbtion of citrate ions on HAp samples prepared by three different methods. Iafisco et al. (2015) discovered that CA and citrates played a key dual role in the apatite crystallization: driving a growth pathway via an amorphous precursor and controlling the size of nanocrystals by the non-classical oriented aggregation mechanism. Additionally, the effects of the CA addition in different amounts (0, 2, 4 and 6 ml) on the structural and dielectric properties of HAp samples prepared in acidic conditions (pH 2) were studied (Kaygili et al. 2015). Furthermore, citrates were found to stabilize both the shape and dimensions of biological apatite crystals in bones (Xie and Nancollas 2010; Hu et al. 2010). All the aforementioned clearly indicates to the complexity of interactions between HAp and CA and the necessity of further experimental investigations.

In present work, considering the above-mentioned key roles of CA combined with the necessity to improve bioactivity of HAp, we prepared four HAp samples in the presence of different CA contents (0, 1, 5 and 10 ml) using the sol–gel approach. We investigated the effects of various amounts of CA on the structure and properties of HAp by means of X-ray diffraction (XRD), Fourier transform infrared (FTIR) spectroscopy, scanning electron microscopy (SEM), energy dispersive X-ray (EDX) spectroscopy techniques. To investigate the influence of CA on the bioactive properties of HAp and to determine the optimum amount of CA, in vitro soaking tests in simulated body fluid (SBF) were performed.

## Materials and methods

### Synthesis

The following chemicals were used in this study. Calcium nitrate tetrahydrate (Ca(NO_3_)_2_·4H_2_O), diammonium hydrogen phosphate ((NH_4_)_2_HPO_4_), CA and ammonium hydroxide (NH_4_OH) were purchased from Sigma-Aldrich and used without further purification. Ultra pure water was used as a solvent. Initially, 400 ml of 0.5 M Ca(NO_3_)_2_ solution and 400 ml of 0.3 M (NH_4_)_2_HPO_4_ solution were prepared in equal quantities by dissolving the necessary amounts of solid reagents in water. Afterwards, both solutions were divided into four equal parts of 100 ml each. To investigate the influence of CA, 0.5 M CA solution was prepared by dissolving solid CA in water and 0, 1, 5 and 10 ml of it were added to the aforementioned portions of Ca(NO_3_)_2_ solutions, respectively. Each beaker with the Ca(NO_3_)_2_ solution was placed onto a magnetic stirrer and heated up to 85 °C. Subsequently, 100 ml portions of 0.3 M (NH_4_)_2_HPO_4_ solutions were poured drop wise into the Ca(NO_3_)_2_ solutions to get the final Ca/P ratio equal to 1.67. In all cases, the solution pH was adjusted to 10 by adding NH_4_OH. In all beakers, gel formation was observed as the result. The gels were kept stirring for 12 h to provide crystal growth, followed by drying at 140 °C for 17 h in an oven and sintering at 1100 °C for 4 h in an electric furnace. White HAp powders were obtained as the result. Taking into consideration the CA contents, the prepared samples were named as CA0-HAp, CA1-HAp, CA5-HAp and CA10-HAp, respectively.

### Structural characterization

The functional groups of the HAp samples compacted into KBr pellets (1.5 mg HAp + 150 mg KBr) were determined using a Fourier transform infrared (FTIR) spectrophotometer (PerkinElmer Spectrum One) in the scanning range of 450–4000 cm^−1^ with 4 cm^−1^ spectral resolution. The crystal structure and phase analyses of the samples were examined by a Bruker D8 Advance X-ray diffraction (XRD) instrument with CuKα radiation (*λ* = 0.15406 nm) generated at 40 kV and 40 mA, and XRD patterns were recorded in the interval of 25–55°. The surface morphology of the samples was investigated using a scanning electron microscope (SEM, JEOL JSM 7001F) equipped with an energy dispersive X-ray (EDX, Oxford Instruments Inca Energy 350) spectrometer operated at an accelerating voltage of 20 kV.

### In vitro tests

To perform in vitro tests, the HAp powders were uniaxially compressed into pellets of 3 mm thick and 12 mm in diameter under the pressure of 10 MPa and then immersed into glass beakers containing 100 ml simulated body fluid (SBF) prepared according to the recipe developed by Kokubo (1991) at 36.5 °C. SBF solutions were renewed every second day. After 2 and 4 weeks soaking, the samples were filtered, washed with distilled water two times and dried. Afterwards, all the aforementioned types of structural analyses (e.g., XRD, FTIR, SEM and EDX) were performed again to observe the effects happened to the samples during their soaking in SBF.

## Results and discussion

**Fig. 1:**
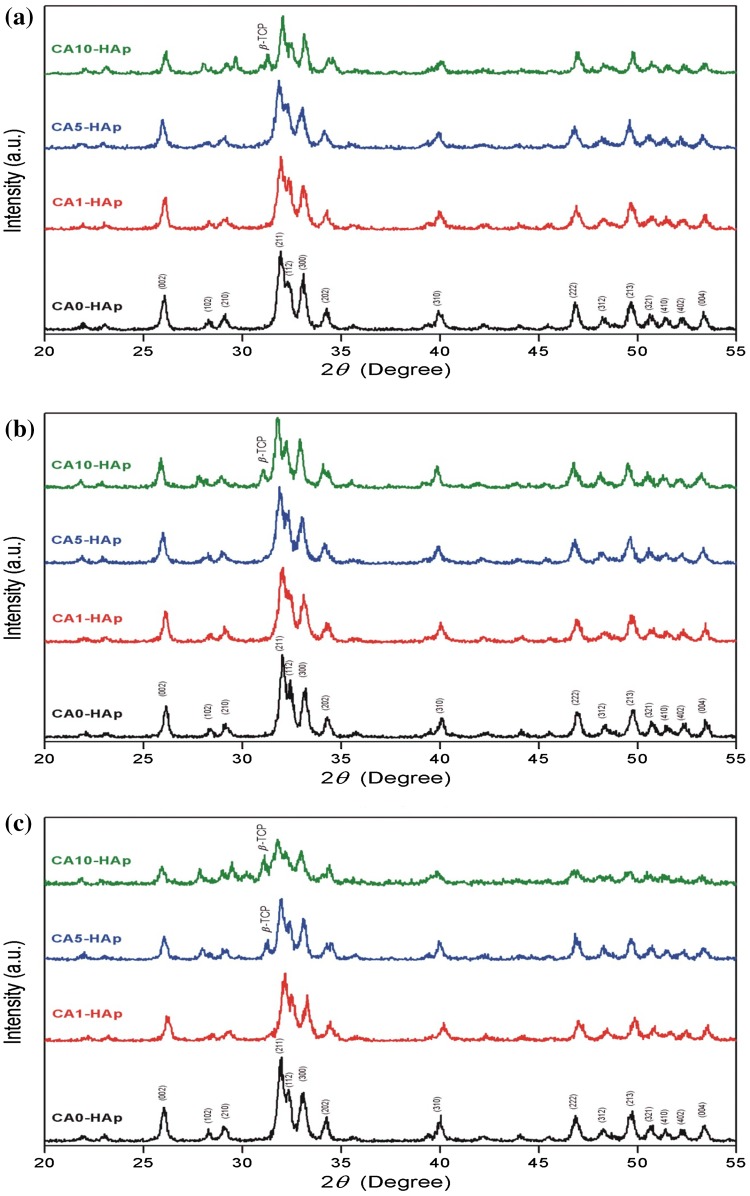
XRD patterns of the HAp samples: **a** before soaking in SBF, **b** after soaking in SBF for 14 days and **c** after soaking in SBF for 28 days

The crystallite dimensions (*D*), unit-cell parameters, crystallinity percentage (*X*_*C*_ %), crystal orientations and phase compositions of the samples were evaluated by XRD. The XRD patterns of the CA-free and CA-containing HAp samples before and after soaking in SBF for 2 and 4 weeks are shown in Fig. 1. For each sample, HAp (JCPDS PDF No. 09–0432) was found to be the major crystal phase. As shown in Fig. 1, CA addition resulted in partial amorphization of HAp, which is seen by intensity decreasing of the major diffraction peaks. Taking into the consideration the well-known chelating properties of CA (Soccol et al. 2006), the presence of CA promoted HAp amorphization by partial consumption of Ca^2+^ ions and, thus, reducing the available amount of Ca below the Ca/P ratio 1.67, which was necessary for the stoichiometric HAp formation. Therefore, in the presence of CA, a non-stoichiometric and poorly crystalline Ca-deficient HAp (Ca_10-*x*_(HPO_4_)_*x*_(PO_4_)_6-*x*_(OH)_2-*x*_) was precipitated. Besides, formation of the secondary phase of β-tricalcium phosphate (β-TCP, JCPDS PDF No. 09–0169) with lower intensities at around 2*θ* = 31.06° was detected for CA10-HAp samples with the highest content of CA, due to a thermal decomposition of the Ca-deficient HAp according to the following equation:

1 $$ {\text{Ca}}_{10 - x} ({\text{HPO}}_{4} )_{x} ({\text{PO}}_{4} )_{6 - x} ({\text{OH}})_{2 - x} \to {\text{Ca}}_{10} ({\text{PO}}_{4} )_{6} ({\text{OH}})_{2} + \beta-{\text{Ca}}_{3} ({\text{PO}}_{4} )_{2} $$

In addition, a presence of β-TCP was also detected in CA5-HAp sample after its soaking in SBF for 4 weeks. Therefore, one can conclude that the presence of CA influenced the formation of the secondary β-TCP phase. In the present study, the formation of the secondary β-TCP phase was observed for CA-containing HAp prepared at basic conditions.

**Fig. 2:**
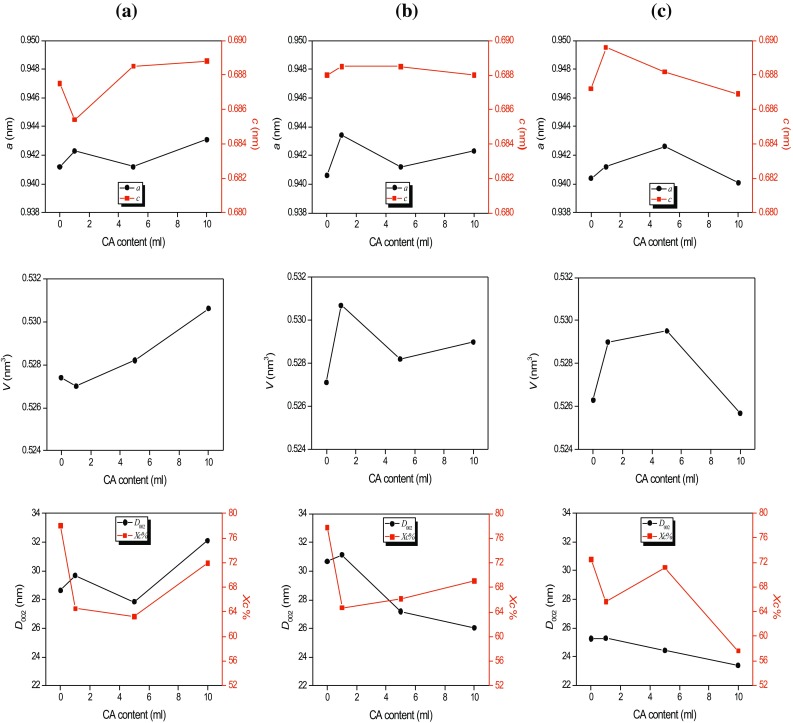
The plots of the unit cell parameters (*a*, *c*), unit cell volume (*V*), crystallite dimensions (*D*_002_) and crystallinity percentage (*X*_*C*_ %) vs. CA content: **a** before soaking in SBF, **b** after soaking in SBF for 14 days and **c** after soaking in SBF for 28 days

The crystallite dimensions (*D*_002_) of the samples along to *c*-axis were evaluated according to the following Scherrer equation using the line broadening of the (002) reflection (Cullity 1978):

2 $$ D_{002} = \frac{0.9\lambda }{{B_{1/2} \cos \theta }} $$

where *λ* is the wavelength of the incident X-rays, *B*_1/2_ is the full width at half maximum (FWHM) and *θ* is the diffraction angle. Additionally, in order to estimate the crystallinity percentage (*X*_*C*_ %) of the samples, the following relation proposed by Landi et al. (2000) was used:

3 $$ X_{C} \% = \left( {1 - \frac{{V_{112/300} }}{{I_{300} }}} \right)\, {\times} \,100$$

where $V_{112/300}$ is the intensity of the hollow between (112) and (300) reflections, and *I*_300_ is the intensity of the (300) diffraction peak. The lattice parameters (*a* = *b* and *c*) and unit cell volume (*V*) of HAp with the hexagonal structure were estimated according to the equations given in the previous study (Kokubo 1991), and these values are mentioned in Table 1. Additionally, the graphs of the lattice parameters of *a* and *c*, unit cell volume (*V*), crystallite dimensions (*D*_002_) and crystallinity percentage (*X*_*C*_ %) as a function of the amount of CA are shown in Fig. 2. By the analyzing the aforementioned table and figure, it can be said that all the calculated parameters related to the crystal structure of HAp were dramatically affected by both CA content and immersion period. All the parameters were found to be changed with increasing of both the CA content and soaking time, but these changes were not gradual. The crystallite dimensions were decreased by stages with increasing soaking time for the samples of CA5-HAp and CA10-HAp. With increasing soaking time in SBF, the values of the crystallinity percentage, the lattice parameter of *a* and the unit cell volume (*V*) were decreased gradually for CA0-HAp and CA10-HAp, while the *X*_*C*_ % increased by stages for CA1-HAp and CA5-HAp, and there was a gradual decrease in the lattice parameter of *c* for CA10-HAp.

**Table 1:** The estimated values of the crystallite dimensions (*D*_002_), crystallinity percentage (*X*_*C*_ %) and unit cell parameters (*a*, *c* and *V*) for all samples before and after soaking in SBF

Sample	*D*_002_ (nm)	*X*_*C*_ %	*a* (nm)	*c* (nm)	*V* (nm^3^)
Before SBF					
CA0-HAp	28.63	78.0	0.9412	0.6875	0.5274
CA1-HAp	29.67	64.5	0.9423	0.6854	0.5270
CA5-HAp	27.84	63.3	0.9412	0.6885	0.5282
CA10-HAp	32.12	71.9	0.9431	0.6888	0.5306
After SBF (14 days)					
CA0-HAp	30.67	77.7	0.9406	0.6880	0.5271
CA1-HAp	31.14	64.7	0.9434	0.6885	0.5307
CA5-HAp	27.19	66.2	0.9412	0.6885	0.5282
CA10-HAp	26.06	69.1	0.9423	0.6880	0.5290
After SBF (28 days)					
CA0-HAp	25.26	72.5	0.9404	0.6872	0.5263
CA1-HAp	25.27	65.7	0.9412	0.6896	0.5290
CA5-HAp	24.43	71.2	0.9426	0.6882	0.5295
CA10-HAp	23.37	57.6	0.9401	0.6869	0.5257

In the previous studies, it was reported that the immersion period in SBF caused the changes in both the lattice parameters (*a* and *c*) and the unit cell volume (*V*) (Hu et al. 2010; Bayraktar and Tas 1999; Kaygili et al. 2014). Therefore, the variations in these parameters are a very good agreement with the reported results in the literature.

**Fig. 3:**
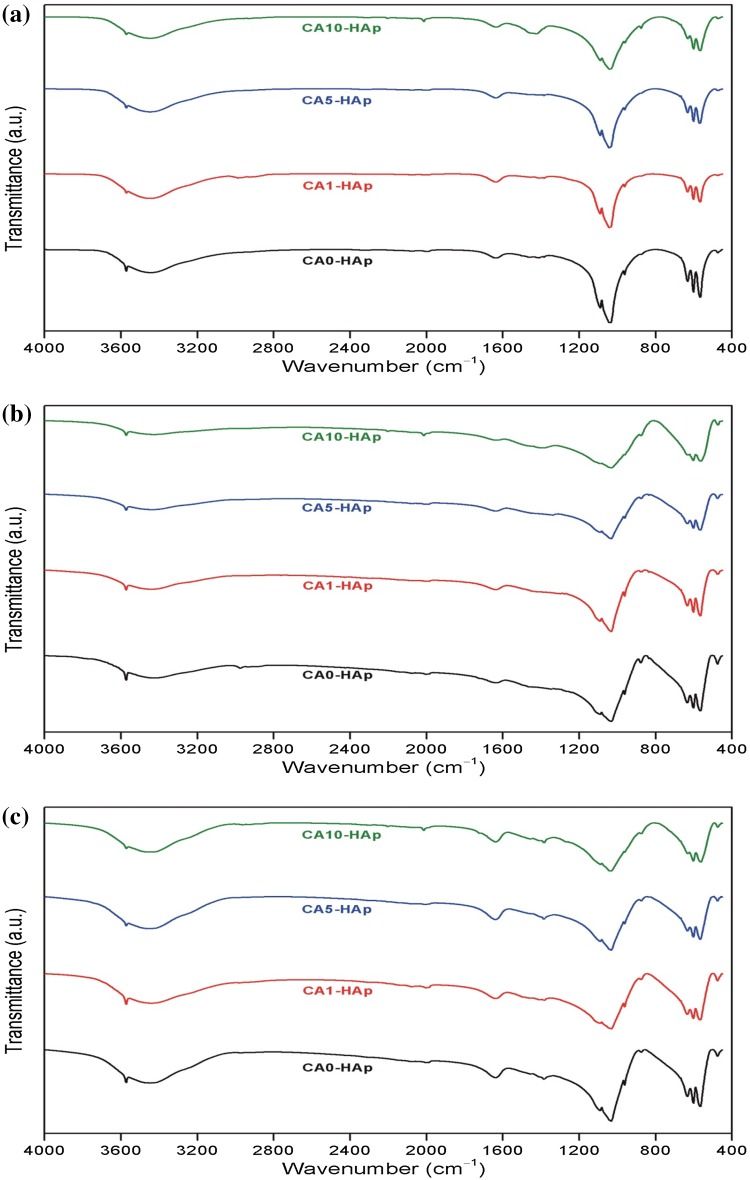
FTIR spectra of the as-prepared HAp samples: **a** before soaking in SBF, **b** after soaking in SBF for 14 days and **c** after soaking in SBF for 28 days

The FTIR spectra of all the samples before and after soaking in SBF for 14 and 28 days are shown in Fig. 3. The bands observed at these spectra verify that each sample contains the functional groups of the phosphate (${\text{PO}}_{4}^{3 - }$) and hydroxyl (${\text{OH}}^{ - }$) belonging to HAp. The bands detected at around 566, 601, 962, 1039 and 1089 cm^−1^ were associated to the phosphate group (Bueno et al. 2014; Okulus et al. 2014; Fahami et al. 2011). The bands corresponding to hydroxyl groups were observed around 631 and 3571 cm^−1^ (Kaygili et al. 2014). The wide absorption bands about 1638 and 3450 cm^−1^ were related to the adsorbed water in the samples and/or in the KBr pellet (Wang et al. 2011; Torabinejad et al. 2014).

**Fig. 4:**
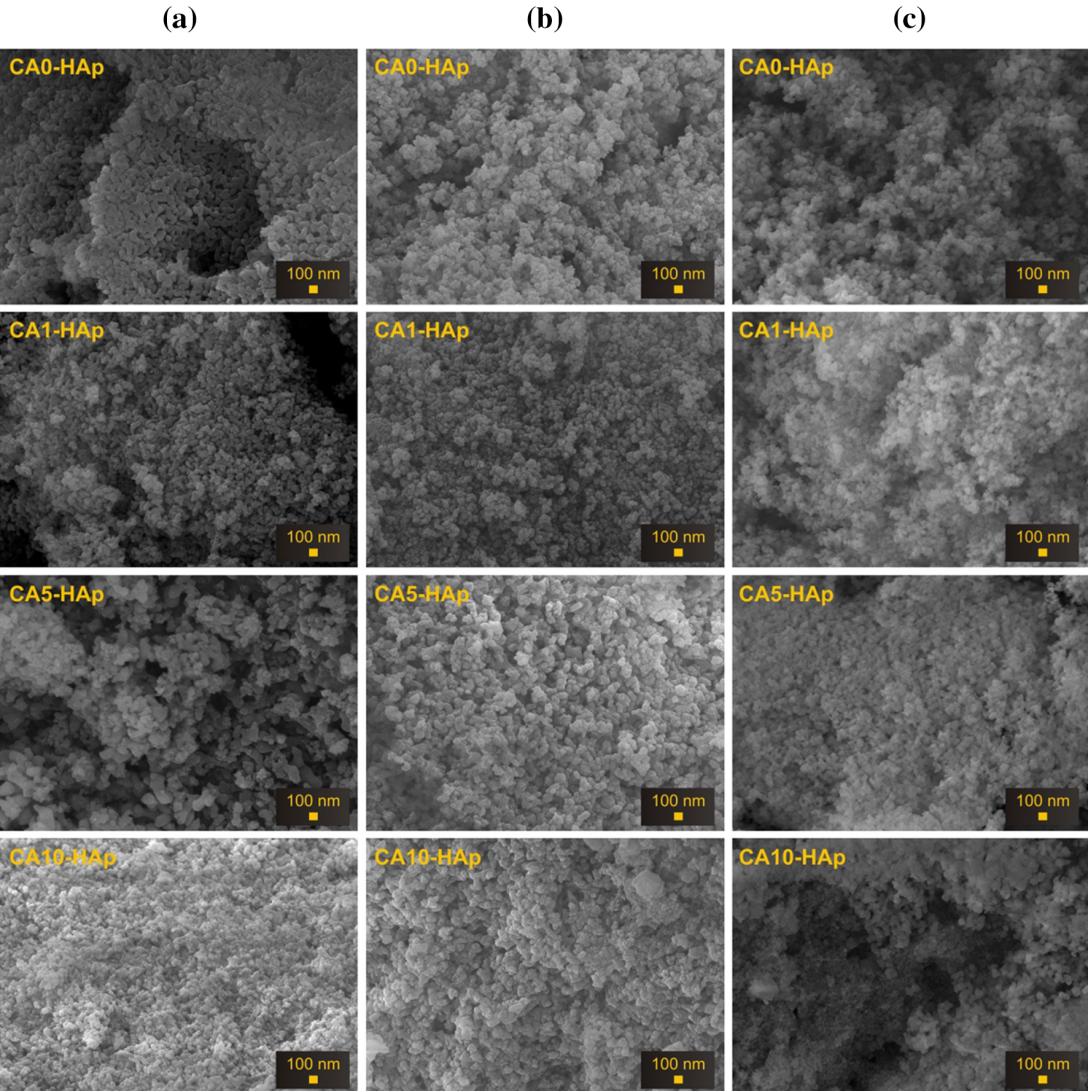
SEM images of the HAp samples soaked in SBF for **a** 0 day, **b** for 14 days and **c** for 28 days

**Fig. 5:**
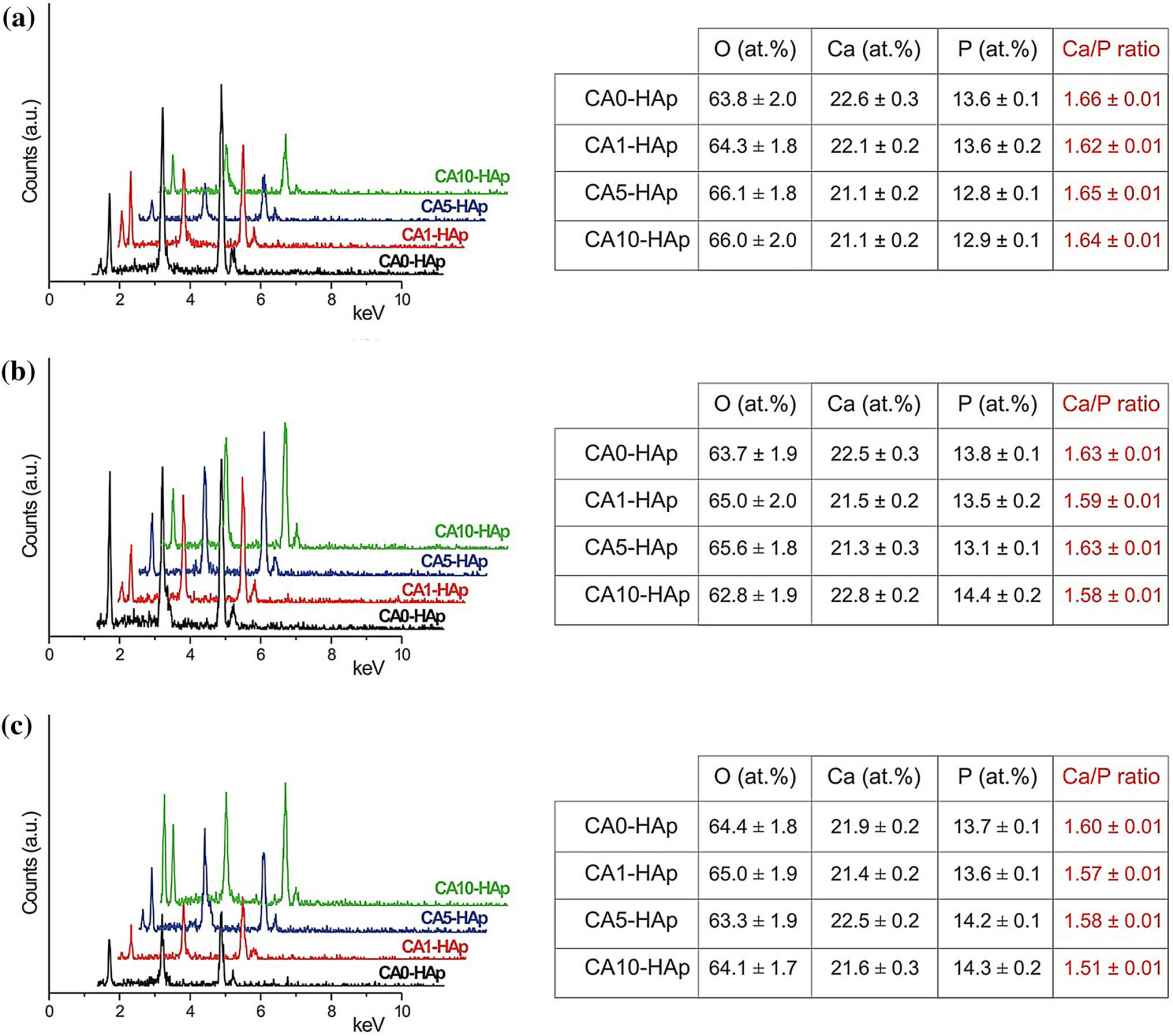
EDX spectra and analysis report of the HAp samples: **a** Before soaking in SBF, **b** after soaking in SBF for 14 days and **c** after soaking in SBF for 28 days

The morphologies of the samples were investigated using SEM, while the elemental analyses of them were performed by EDX. The SEM micrographs and the results of the EDX analysis for both the CA-free and CA-containing samples, performed both before and after soaking in SBF for 14 and 28 days are presented in Figs. 4 and 5, respectively. It is clearly seen that all the samples were composed of tiny crystals, which were generally smaller than 100 nm (Fig. 4). There was almost no change in their morphology with increasing amount of CA and immersion time. The microporosity was observed for all the samples, and this is one of the most desired properties for a clinical reconstructive material (Sopyan et al. 2007). Moreover, it was reported that the microporous surfaces may modulate the adsorption of proteins from serum, as well as the adhesion and proliferation of human bone cells (Rouahi et al. 2006). On account of this, it can be said that CA-assisted HAp samples synthesized by sol–gel route can be used as an implant material for biomedical applications. For each sample, the elements of Ca, P and O were detected from EDX, while no impurities were detected (Fig. 5). The presence of these elements and their Ca/P molar ratios confirmed that HAp was always formed. Both the immersion time and the amount of CA affected the elemental composition, as well as these factors influenced the Ca/P ratio. Furthermore, with increasing soaking time the Ca/P ratio was gradually decreased for each sample. This decrease was due to precipitation of a non-stoichiometric Ca-deficient HAp from SBF, which is in a good agreement with the earlier reports on the subject (Kaygili et al. 2014, 2015; Hu et al. 2010; Wan et al. 2006). The soaking results in SBF revealed that the maximum changes in the Ca/P ratio were found for the CA10-HAp samples (Fig. 5), which pointed out to the maximum amount of non-stoichiometric Ca-deficient HAp precipitation and, therefore, the highest bioactivity of these samples.

## Conclusions

Both CA-free and CA-containing HAp samples, which were composed of tiny crystals smaller than 100 nm, were easily prepared in basic conditions using the sol–gel technique, and the effects of CA content and immersion time in SBF on their structural properties including the phase composition, crystallinity, cell parameters, chemical structure, morphology and elemental composition were investigated in details by the XRD, FTIR, SEM and EDX. The CA content was found to affect the in vitro performance of the HAp samples because changes in the structural properties of the samples were different for the immersion periods of 14 and 28 days in SBF. Although the samples’ morphology was found to be affected by neither the amount of CA nor the soaking time in SBF, the soaking results revealed that the maximum changes in the Ca/P ratio were found for the HAp samples prepared in the presence of the highest amounts of CA, which pointed out to the highest bioactivity of these samples. Considering the above-mentioned conclusions, it can be said that the CA-assisted nano-sized HAp powders may be a very good nominate for biomedical applications.
